# Identification of a three-miRNA signature as a novel prognostic model for papillary renal cell carcinoma

**DOI:** 10.1186/s12935-020-01398-2

**Published:** 2020-07-16

**Authors:** Ge Li, Haifan Yang, Yong Cheng, Xin Zhao, Xu Li, Rui Jiang

**Affiliations:** Department of Urology, The Affiliated Hospital of Southwest Medical University, No. 25 Taiping Road, Jiangyang District, Luzhou, 646000 China

**Keywords:** Papillary renal cell carcinoma, miRNAs, Risk score, Prognosis

## Abstract

**Background:**

Papillary renal cell carcinoma (pRCC) accounting for near 20% of renal cell carcinoma is the second most common histological subtype. MiRNAs have been demonstrated to played significant roles on predicting prognosis of patients with tumors. An appropriate and comprehensive miRNAs analysis based on a great deal of pRCC samples from The Cancer Genome Atlas (TCGA) will provide perspective in this field.

**Methods:**

We integrated the expression of mRNAs, miRNAs and the relevant clinical data of 321 pRCC patients recorded in the TCGA database. The survival-related differential expressed miRNAs (sDEmiRs) were estimated by COX regression analysis. The high-risk group and the low-risk group were separated by the median risk score of the risk score model (RSM) based on three screened sDEmiRs. The target genes, underlying molecular mechanisms of these sDEmiRs were explored by computational biology. The expression levels of the three sDEmiRs and their correlations with clinicopathological parameters were further validated by qPCR.

**Results:**

Based on univariate COX analysis (*P *< 0.001), eighteen differential expressed miRNAs (DEmiRs) were remarkably related with the overall survival (OS) of pRCC patients. Three sDEmiRs with the most significant prognostic values (miR-34a-5p, miR-410-3p and miR-6720-3p) were employed to establish the RSM which was certified as an independent prognosis factor and closely correlated with OS. In the verification of clinical samples, the overexpression of miR-410-3p and miR-6720-3p were detected to be associated with the advanced T-stages, while miR-34a-5p showed the reversed results.

**Conclusion:**

The study developed a RSM based on the identified sDEmiRs with significant prognosis prediction values for pRCC patients. The results pave the avenue for establishing and optimizing a reliable and referable risk assessing model and provide novel insight into the researches of biomarkers and clinical treatment strategies.

## Background

With the increasing morbidity, papillary renal cell carcinoma (pRCC) accounting for near 20% of renal cell carcinoma (RCC), has transcended into the second most common histological subtype [1, 2]. Following the new WHO classification of RCC subtypes, pRCC was identified to be the type 1 and 2 on basis of the histopathological morphology [3, 4]. Characterized by the multifarious biological behaviors of nonreactivity or invasiveness, pRCC usually emerged as the focal or heterogeneous multifocal tumor [5]. In the past few years, a growing body of researchers have been focusing on the pRCC to discover the pathogenesis and pathological features and further explore the effective treatments, of which targeted therapies and immunotherapies are attracting increasing attentions [6, 7].

Although Food and Drug Administration (FDA) has approved certain drugs of immunotherapy and targeted therapy, and some of which gained encouraging outcomes for only a few subgroups of patients with pRCC, a wider range of pRCC patients remain in desperate need of the more ideal and promising treatment strategies [8, 9]. From another perspective, identification of more valuable biomarkers to predict the therapy response rate and prognosis will contribute to beforehand distinguish personalized patients with satisfied sensitivities and then improve the whole therapeutic efficiency. Therefore, we intended to explore the appropriate indictors to achieve individualized treatments.

Consisting of abundant immune molecules and other regulating cytokines, tumor microenvironment (TME) closely associating with the tumor immune response processes, is the crucial concern of discovering biological markers [10, 11]. With the increasingly thorough insights of the vital effects of genetics and genetic modification approaches on tumor behaviors and prognosis, researchers have been identifying the rising numbers of genetic markers including certain coding genes and non-coding genes such as long non-coding RNA (lncRNAs) and microRNAs (miRNAs) in TME, however, their potentials for the prediction of prognosis are awaiting to be adequately elucidated [12–14].

As a kind of small endogenously expressed RNA molecules with approximately 17–23 nucleotides in length, miRNAs play indispensable roles on the genes posttranscriptional regulations including messenger RNAs (mRNAs) cleavage and protein translation [15, 16]. Furthermore, the stability and testability of miRNAs in various biological samples remarkably improve the superiorities of being biomarkers [17, 18]. Therefore, a growing number of miRNAs contributing to the diagnosis, treatment and prognosis prediction were identified in different tumors recently. Nishibeppu et al. highlighted the predicting role of miR-1229-3p, with the higher expression levels in gastric cancer patients of poorer prognoses [19]. Weiss et al. established a four-miRNAs model to predict the poor outcomes with the large tumor volume and vessel invasion in hepatoblastoma [20]. However, the roles of miRNAs on the prognosis prediction of pRCC patients haven’t been discovered, besides, fewer studies focused on exploring the target genes of IRmiRs with significances of prognosis forecast.

Therefore, we designed the study to discover and validate the roles of miRNAs on clinical prognosis prediction of pRCC through establishing a more personalized and accuracy risk predicting model. Furthermore, the clinical relevance of the miRNAs and the interactions of their target genes were also detected. The ultimate aim of the study is to offer the guiding light for clinical decision.

## Methods

### Clinical renal samples

28 pRCC tissues (type 1 and type 2) and adjacent tissues were acquired from the patients who accepted operation in The Affiliated Hospital of Southwest Medical University from September 2018 to December 2019. The acquired samples were frozen in liquid nitrogen immediately and then stored at − 80 °C until miRNAs extraction.

### Data download and analysis of differential mRNAs and differential miRNAs

A series of transcriptome RNA-sequencing and miRNA data of pRCC samples including type 1 and type 2 were downloaded from the TCGA data portal (https://portal.gdc.cancer.gov/), which contained data from 32 non-tumor tissues and 389 pRCC samples. These data were updated on May 7, 2020. Clinical data about these patients were downloaded and extracted (the OS of patients ≤ 30 days were excluded because of these patients probably died of unpredictable factors). RNA-seq and miRNAs results were combined into matrix files by the use of a merge script in the Perl language (http://www.perl.org/). The R software limma package (https://bioconductor.org/packages/release/bioc/html/limma.html) was used to screen for differentially expressed genes in tumor and adjacent non-tumor tissues. We present all differential mRNAs and miRNAs analysis data with the screening value of “FDR < 0.05, log_2_| FC | > 1 and P < 0.05”. The differential mRNAs and miRNAs were prepared to the subsequent study.

### Survival-related DEmiRs

Differentially expressed miRNAs (DEmiRs) correlated with survival in patients with pRCC were verified as survival related DEmiRs (sDEmiRs). Univariate COX analysis was employed to screen sDEmiRs (*P *< 0.05). Hazard ratio (HR) was utilized to specified sDEmiRs into protective and deleterious parts. These sDEmiRs were identified for the subsequent study.

### Establishment of the risk score model (RSM)

The sDEmiRs were analyzed by the multivariate analysis, and three sDEmiRs regarded as the independent prognostic indicators were recruited to develop the RSM. In order to investigate the clinical prognosis, pRCC patients were further divided into the high-risk group and the low-risk group on the basis of the median risk score of RSM. The median risk score was regarded as the cutoff point. RSM was established by the expression data multiplied by Cox regression coefficients. The formula was as followed, [Expression levels of miR-34a-5p * (−0.6926312)] + [Expression levels of miR-410-3p * (0.4567728)] + [Expression levels of miR-6720-3p * (0.1965725)]. The values of RSM were utilized to evaluate multiplied subtypes of pRCC patients.

To further explore the significances of the sDEmiRs, we analyzed the correlation of the RSM and clinicopathologic features, of which the “TNM staging method” is regarded as the most common method to evaluate the tumor status. The maximum tumor diameter and tumor invasion extent were used to divide the T-stage, with the larger tumor sizes and more extensive invasion in the more advanced T stages. “N-stage” reflected the lymph node metastasis status, with more metastatic lymph nodes in the more advanced N stages. “M-stage” was distinguished based on the distant metastasis conditions, and the advanced M stages probably represented the poor tumor outcomes. In addition, “stage” was a comprehensive way combining with T-stage, N-stage and M-stage to separate pRCC patients from I, II, III and IV stage.

### Real-time quantitative PCR

Total RNA was extracted from pRCC tumor and adjacent tissues by TRIzol (Invitrogen). RNA was reverse-transcribed to cDNA by the PrimeScript RT reagent kit (TaKaRa, Osaka, Japan). The reaction steps were as follows: 37 °C for 15 min and 85 °C for 5 s. The quantitative polymerase chain reaction (qPCR) was performed on an ABI 7500 Real-Time PCR System (Applied Biosystems) utilizing a SYBR Green assay (TaKaRa). The reaction cycling conditions (95 °C for 30 s, 40 cycles of 95 °C for 5 s, and 60 °C for 34 s) were carried out; primer sequences are shown in Table 1. The relative quantification values of miRNAs were standardized to U6 using the 2^−ΔCt^ method. Three replicate assays were performed per cDNA sample.

**Table 1:** The primer sequences of hsa-miR-34a-5p, hsa-miR-410-3p and hsa-miR-6720-3p

hsa-miR-34a-5p	F primer (5′–3′)	AACACGCTGGCAGTGTCTTA
	R primer (5′–3′)	GTCGTATCCAGTGCAGGGT
	RT (5′–3′)	GTCGTATCCAGTGCAGGGTCCGAGGTATTCGCACTGGATACGACACAACC
hsa-miR-410-3p	F primer (5′–3′)	ATGCGCGCAATATAACACAGA
	R primer (5′–3′)	GTCGTATCCAGTGCAGGGT
	RT (5′–3′)	GTCGTATCCAGTGCAGGGTCCGAGGTATTCGCACTGGATACGACACAGGC
hsa-miR-6720-3p	F primer (5′–3′)	AATATTACGCGCCTGCAGGA
	R primer (5′–3′)	GTCGTATCCAGTGCAGGGT
	RT (5′–3′)	GTCGTATCCAGTGCAGGGTCCGAGGTATTCGCACTGGATACGACTCTACC
U6	F primer (5′–3′)	CTCGCTTCGGCAGCACA
	R primer (5′–3′)	AACGCTTCACGAATTTGCGT
	RT (5′–3′)	AAAATATGGAACGCTTCACGAATTTG

*F primer* forward primer, *R primer* reverse primer, *RT* reverse transcription

### The selection of target genes and bioinformatics analysis

The target genes were selected by the databases of TargetScan (http://www.targetscan.org/vert_72/), miRTarBase (http://mirtarbase.mbc.nctu.edu.tw/php/index.php) and miRDB (http://mirdb.org/). And the filter standard for a target gene was no less than two databases supported it. In order to explore the interaction between these target genes, a PPI network based on the data was acquired on the STRING online database (https://string-db.org/). PPI networks were employed to show the relationships between these target genes. The standard for a core gene was no less than five node degrees. Cytoscape software version 3.7.2 was used to show PPI results. Functional enrichment analysis was performed through the Gene Ontology (GO) and Kyoto Encyclopedia of Genes and Genomes (KEGG) pathways to explore the underlying molecular mechanisms of differential IRGs. GO and KEGG pathways were on the basis of R software packages of cluster profiler, org.Hs.e.g.db, and enrichplot.

### Statistical analysis

In order to verify the prognosis, the ROC curve was drawn by the survival ROC package of the R software. The abscissa is the specificity (false positive rate), and the ordinate represents the sensitivity (true positive rate). Univariate Cox regression analysis, Pearson correlation analysis and multivariate regression analysis were utilized to confirm the sDEmiRs. Kaplan–Meier curve was employed to estimate the OS of the high-risk group and the low-risk group of pRCC patients. All statistical analysis was conducted by SPSS21.0 software (SPSS Inc, Chicago, IL) and GraphPad Prism5 (GraphPad Software Inc, La Jolla, CA). Variations in clinical parameters were determined via independent *t* test. *P *< 0.05 was considered statistically significant.

## Results

### Differential expression of mRNA and miRNA

**Fig. 1:**
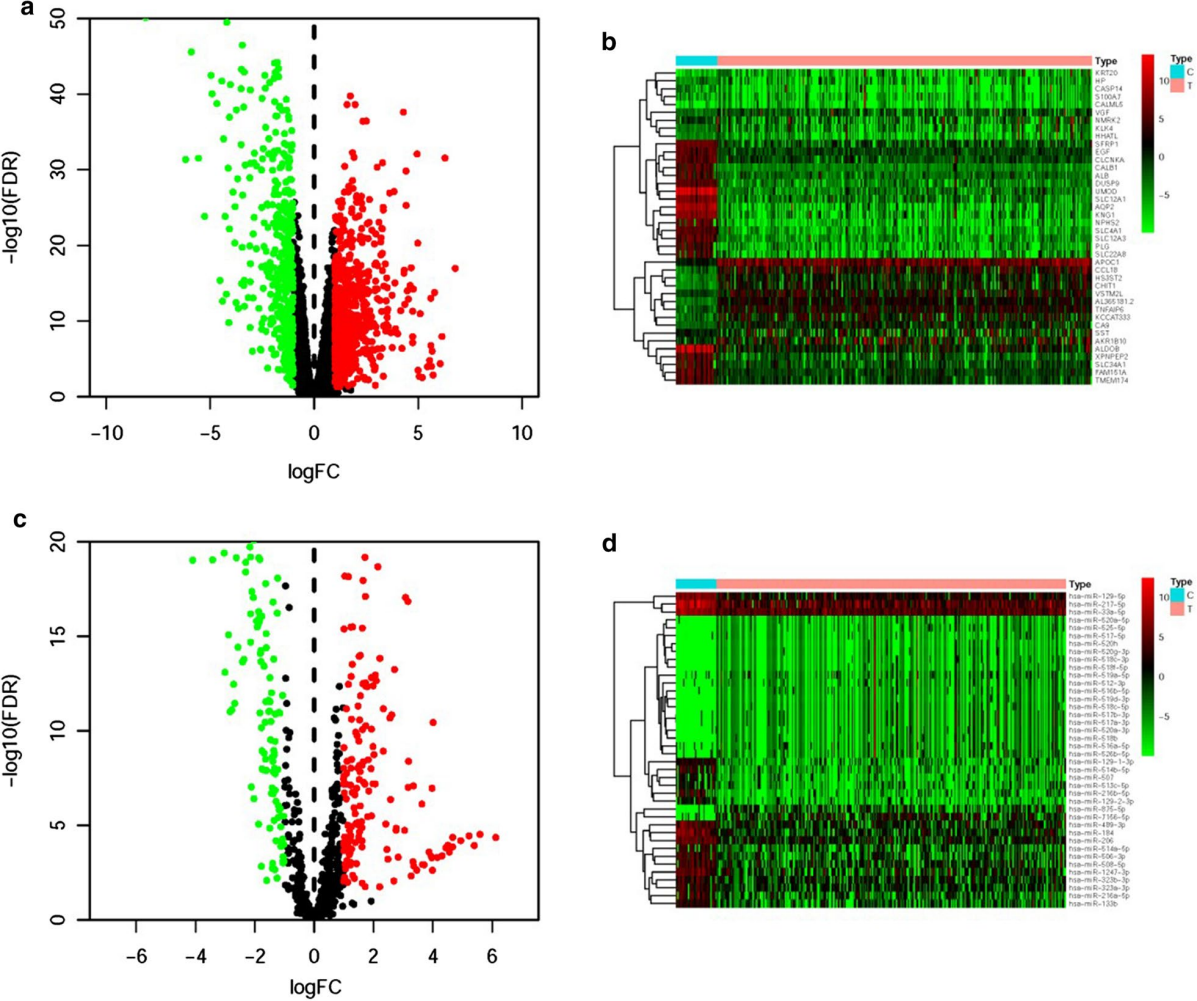
Differentially expressed pRCC mRNAs and miRNAs. Volcano plot (**a**) and heatmap (**b**) illustrated the differentially expressed mRNAs between tumor tissues and adjacent tissues. The differentially expressed miRNAs were illustrated in the volcano plot (**c**) and heatmap (**d**). The green dots represented the downregulated genes; the red dots represented the significantly upregulated genes, and the black dots represented the genes without differential expression. FDR < 0.05, log_2_ | FC | > 1 and *P* < 0.05

1244 differentially expressed pRCC genes were screened by limma algorithm, of which 462 were down-regulated and 782 were up-regulated (Fig. 1a). Next, the 20 most up-regulated and down-regulated genes were respectively identified by the values of log_2_∣FC∣ and the heatmap was illustrated in Fig. 1b. We further confirmed 308 DEmiRs, including 142 down-regulated and 164 up-regulated miRNAs (Fig. 1c). And the most 20 up-regulated and down-regulated miRNAs were shown in the heatmap (Fig. 1d).

### The relationships between DEmiRs and prognosis

**Fig. 2:**
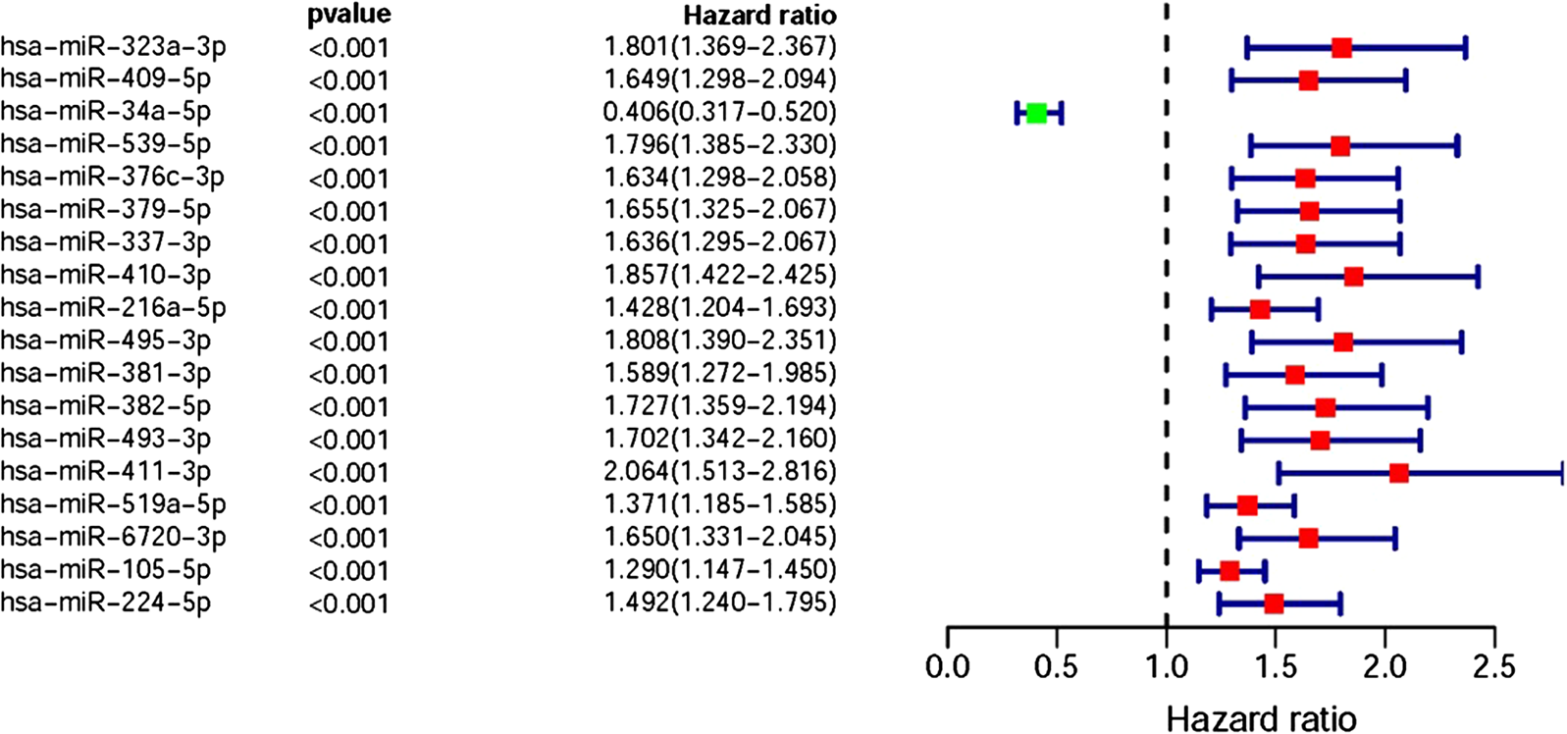
Survival-related DEmiRs. Forest plot of hazard ratios showed the survival-related values of DEmiRs (miR-323a-3p, miR-409-5p, miR-34a-5p, miR-539-5p, miR-376c-3p, miR-379-5p, miR-337-3p, miR-410-3p, miR-216a-5p, miR-495-3p, miR-381-3p, miR-382-5p, miR-493-3p, miR-411-3p, miR-519a-5p, miR-6720-3p, miR-105-5p and miR-224-5p). Red parts represented the upregulated sDEmiRs, and green parts represented the downregulated sDEmiRs

**Fig. 3:**
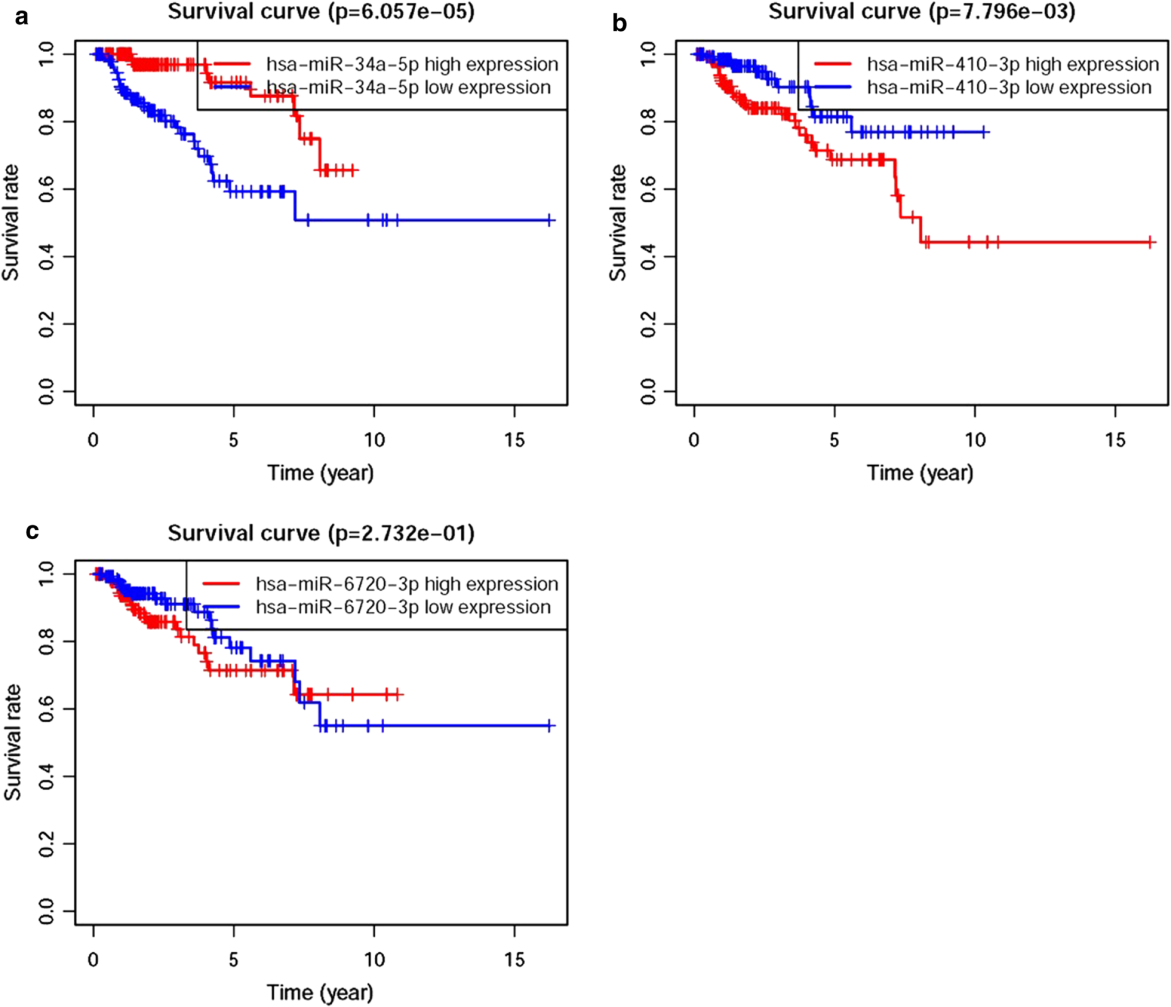
Survival curve of pRCC patients. Kaplan‐Meier survival curve of OS among pRCC patients based on TCGA pRCC data. The higher expression levels of miR-410-3p (**b**) and miR-6720-3p (**c**) were correlated with the poor prognosis. The lower expression level of hsa-miR-34a-5p (**a**) was associated with the poor prognosis

Based on COX Regression model, we screened 18 DEmiRs which were closely associated with the prognosis of patients with pRCC (sDEmiRs), such as miR-323a-3p, miR-409-5p, miR-34a-5p, miR-539-5p, miR-376c-3p, miR-379-5p, miR-337-3p, miR-410-3p, miR-216a-5p, miR-495-3p, miR-381-3p, miR-382-5p, miR-493-3p, miR-411-3p, miR-519a-5p, miR-6720-3p, miR-105-5p and miR-224-5p. And the relationships between these sDEmiRs and prognosis were illustrated in the forest map, in which miR-34a-5p showed a reverse trend to that of other miRNAs, with the negative correlation with the poor clinical outcomes (Fig. 2). In order to establish the risk score model later, three sDEmiRs (miR-34a-5p, miR-410-3p and miR-6720-3p) with significant statistical difference were identified through the multivariate COX analysis (Additional file 1: Table S1). Then, the survival curve of the three sDEmiRs showed that the higher expression level of miR-34a-5p was related with the longer OS, but the miR-410-3p and miR-6720-3p showed negative correlations with OS (Fig. 3a–c).

### Clinical characteristics of the high-risk group and the low-risk group

**Fig. 4:**
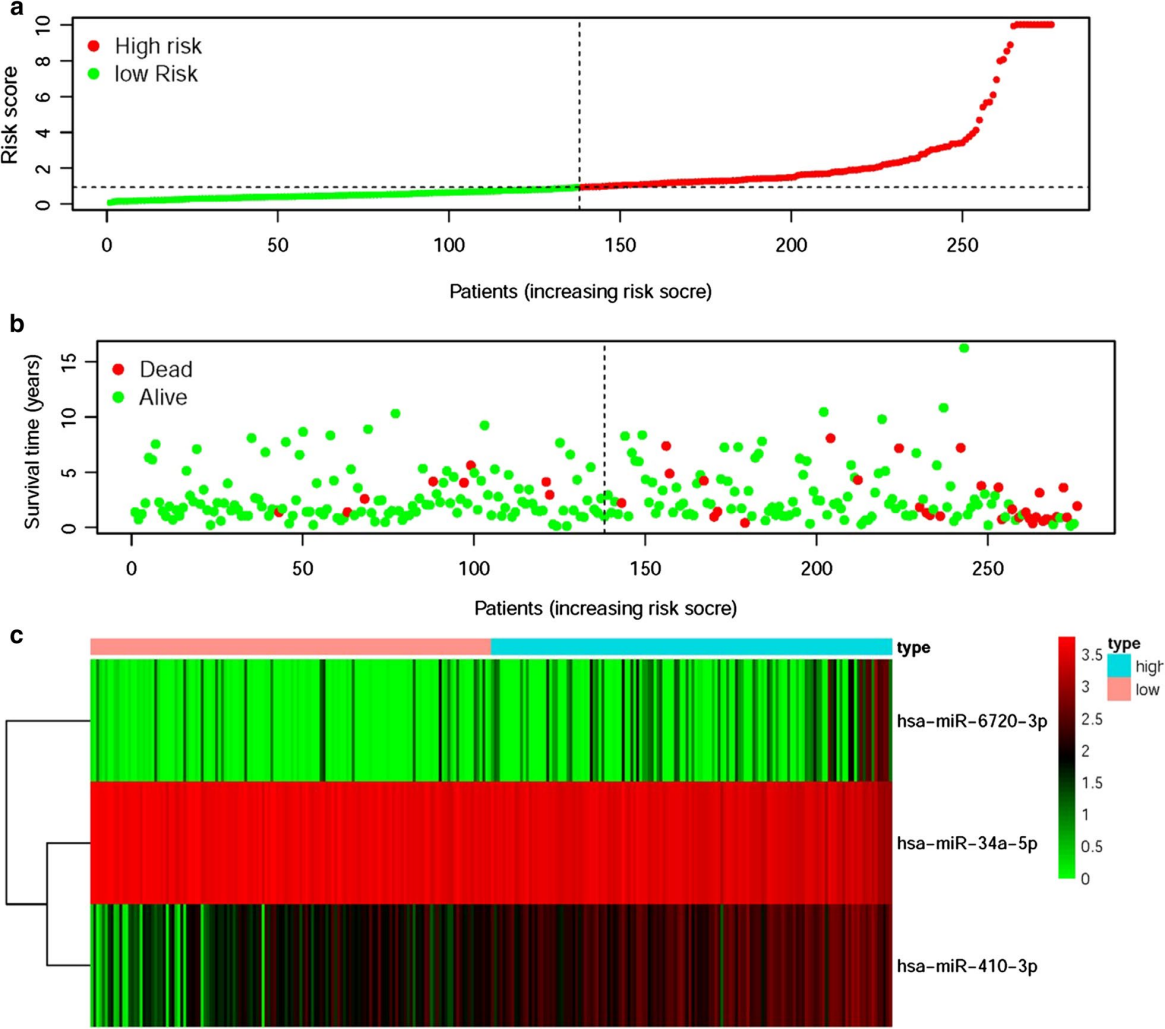
Risk score model (RSM) was established based on sDEmiRs. The risk score distribution in the high-risk group and the low-risk group (**a**). Survival status of the high-risk group and the low-risk group (**b**). The heatmap of the expression levels of sDEmiRs included in the RSM (**c**). In the heatmap, the red parts represented up-regulation, the green parts represented down-regulation, and black parts represented no difference

**Fig. 5:**
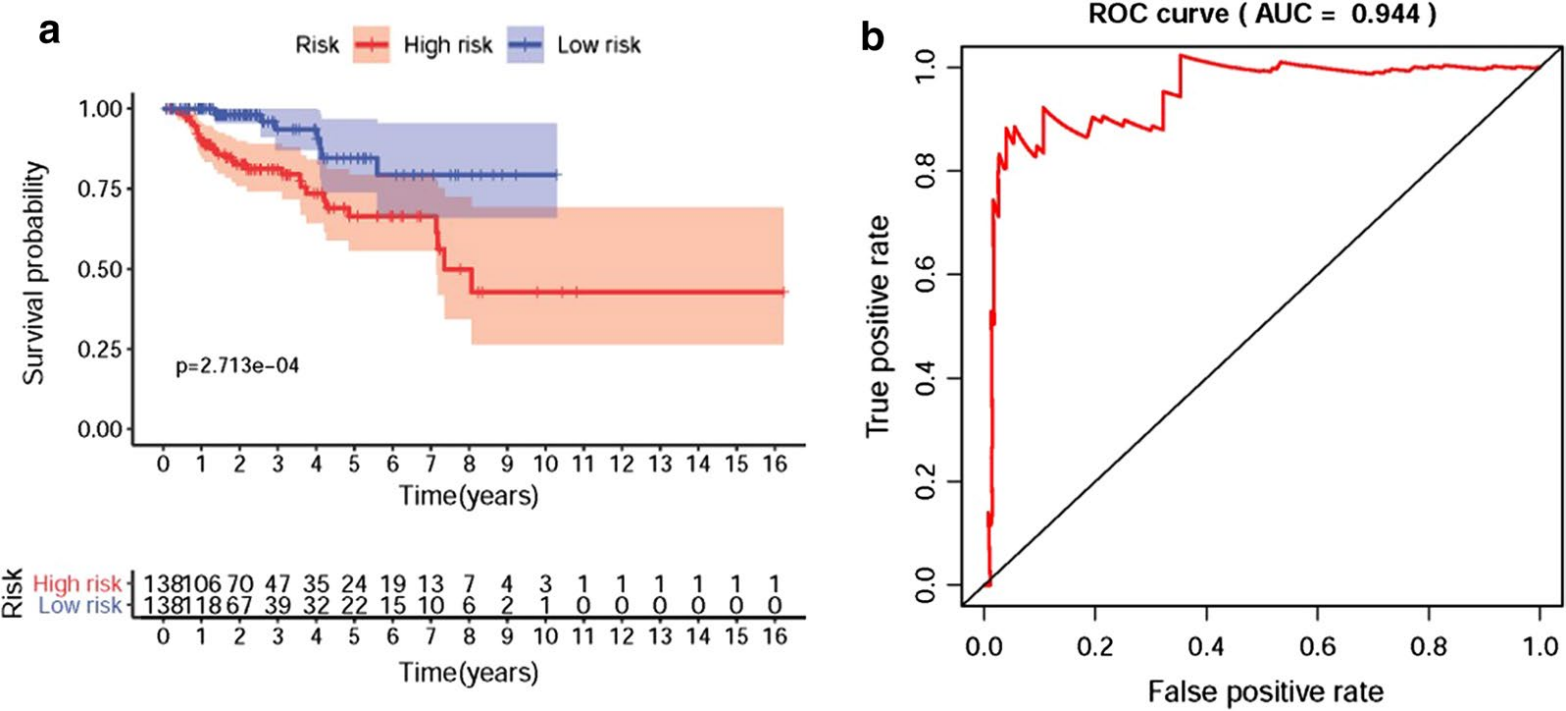
The survival curve of RSM and receiver operating characteristic (ROC) curve. Kaplan‐Meier survival curve of OS in the low-risk group (blue line) and the high-risk group (red line) (**a**). The high-risk group showed the poor prognosis. ROC curves indicated the prognostic value of the independent prognostic factors (**b**). The AUC was 0.944

The selected 3 sDEmiRs among the 18 sDEmiRs were employed to establish RSM, by which the pRCC samples were divided into the high-risk group and the low-risk group (Fig. 4a). The median risk score was the cutoff point. The mortality rate remarkably increased with the higher risk score (Fig. 4b). The five-year survival rates of the high-risk group and the low-risk group were 66.4% and 84.6%, respectively. With the rise of the risk score, the expression levels of miR-6720-3p and miR-410-3p were enhanced, while there was no remarkable difference in the expression level of miR-34a-5p (Fig. 4c). The survival of the low-risk group was significantly longer than that of the high-risk group (Fig. 5a). To detect the accuracy of the model, the ROC curve was employed, and the AUC of the ROC curve was 0.944, suggesting the satisfied accuracy and the great potential of RSM based on the 3 sDEmiRs in survival prediction (Fig. 5b).

### The clinical application of the RSM and the relationships between the RSM and clinical features

**Fig. 6:**
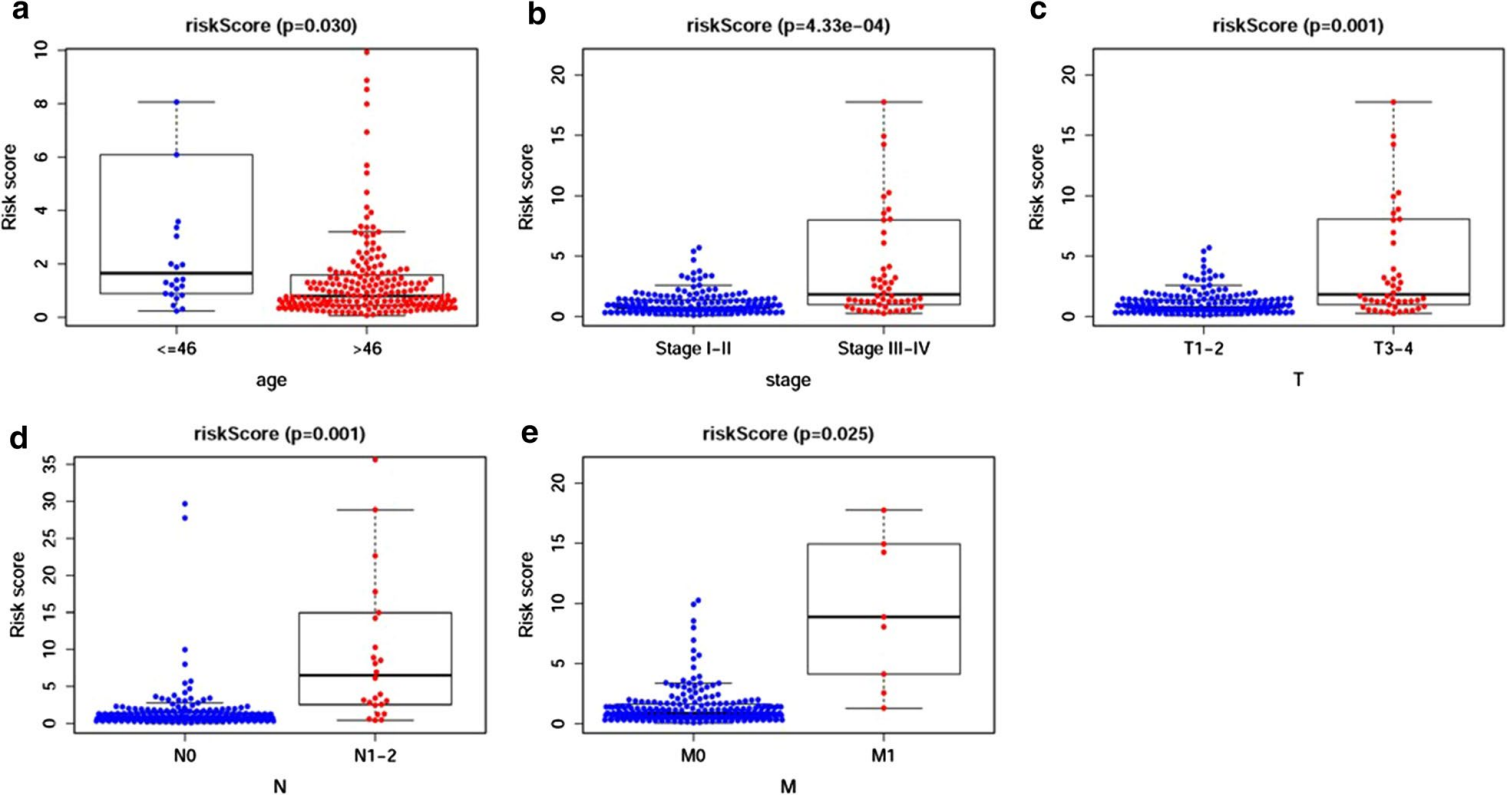
The relationships between the RSM and the clinical features. The relationships between the RSM and age (**a**), tumor stage (**b**), T-stage (**c**), N-stage (**d**) and M-stage (**e**). The older patients (**a**), and patients with earlier stage (**b**), earlier T-stage (**c**), earlier N-stage (**d**) and earlier M-stage (**e**) got the significantly lower risk score based on TCGA clinical data (0 = Female patients; 1 = Male patients)

To explore the relevance of the sDEmiRs and clinical features of pRCC, we then analyzed the correlation between the risk score and the clinical characteristics including age, stage, T-stage, N-stage and M-stage. We found the younger patients (Fig. 6a), and patients with advanced stages (Fig. 6b), advanced T-stages (Fig. 6c), advanced N-stages (Fig. 6d) and advanced M-stages (Fig. 6e) got the remarkably higher risk scores. The aforementioned results suggested the sensitivity of the RSM for some clinical characteristics, and further corroborated the clinical application value of the model to a certain extent. Next, we intended to analyze the relationship between the compositions of the RSM and the demographic characteristics including age and gender. As shown in Additional file 2: Figure S1, only did miR-34a-5p showed the significant correlation with the demographic characteristics and the older patients obtained the higher expression level of miR-34a-5p.

### The relationship between the three sDEmiRs and clinical characteristics

**Fig. 7:**
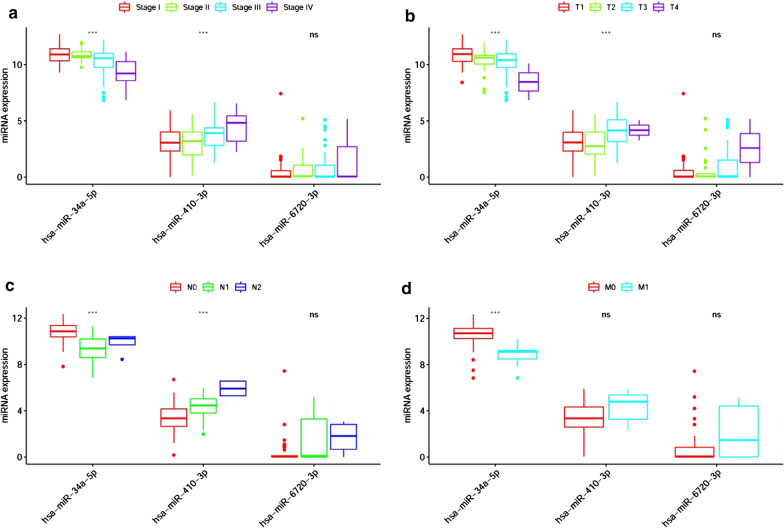
The relationships between the sDEmiRs and clinical features. The expression level of miR-34a-5p was decreased with the more advanced stage (**a**), T-stage (**b**), N-stage (**c**) and M-stage (**d**) based on TCGA pRCC data. The expression level of miR-410-3p was increased with the more advanced stage (**a**), T-stage (**b**) and N-stage (**c**) based on TCGA pRCC data (***P  < 0.001; **P  < 0.01; *P  < 0.05)

To further clarify the significances of the three sDEmiRs, we respectively explored their relationships with clinical characteristics. As illustrated in Fig. 7, We found the expression levels of miR-34a-5p was gradually decreased in patients with the more advanced stages, T-stages, N-stages and M-stages. However, the miR-410-3p showed the reversed variations, with the enhancement in patients with the more advanced stages, T-stages and N-stages. The distinguished correlations of these sDEmiRs with clinical characteristics provide a novel method to predict the tumor staging.

To further verify whether the risk score was available to be the independent prognostic factor, we performed the univariate and multivariate Cox regression analysis. As shown in Table 2, the risk score, age, stage, N-stage and M-stage were closely related to OS in the univariate Cox regression analysis, while the results of the multivariate Cox regression analysis showed the risk score, gender, stage, T-stage and M-stage have the potential for predicting OS. These results reminded us of the feasibility and significance of the risk score to be an independent prognostic factor.

**Table 2:** Univariate and multivariate analysis of pRCC

Variables	Univariate analysis				Multivariate analysis			
	HR	HR 95% low	HR 95% high	P value	HR	HR 95% low	HR 95% high	P value
Age	0.959064	0.920145	0.999629	0.041201	0.966014	0.916583	1.018112	0.196992
Gender	1.551893	0.512361	5.212566	0.568864	0.005817	0.001089	0.031071	1.733e−09
Stage	2.461577	1.397805	4.304486	0.005431	17.28874	6.481088	46.11887	1.246e−08
T-stage	1.829788	0.931084	3.318484	0.098979	0.342463	0.117590	0.997367	0.049437
M-stage	70.69564	8.287564	609.8372	0.000112	24.11689	2.639592	220.3463	0.004804
N-stage	2.206308	1.093178	4.605738	0.033691	0.270470	0.059089	1.238026	0.092018
Risk score	1.035608	1.003836	1.068387	0.027751	0.928709	0.889633	0.969501	0.000745

*HR* Hazard Ratio

### The target genes of the three sDEmiRs target genes and their interactions

**Fig. 8:**
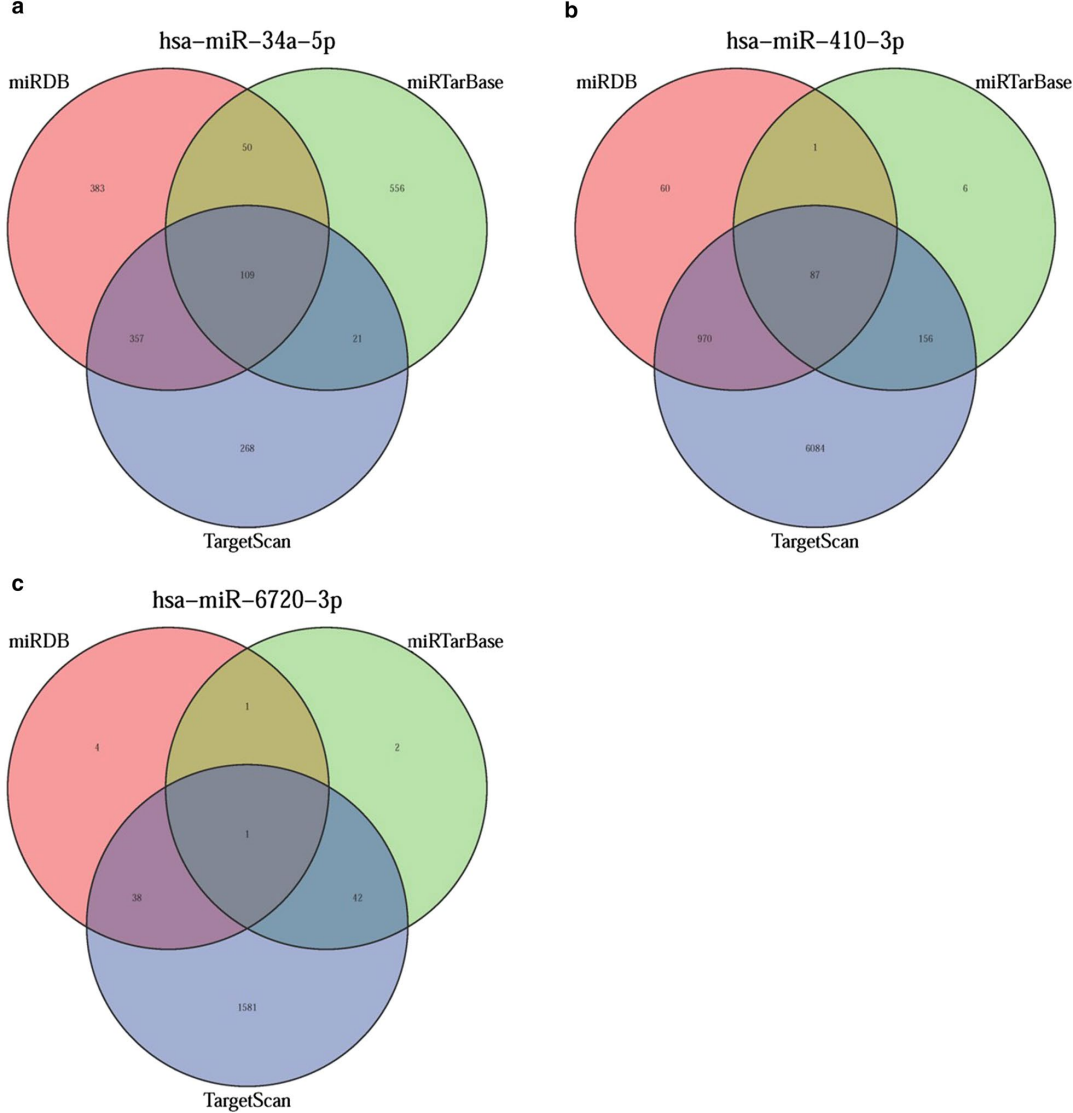
The Venn diagram of the sDEmiRs target genes. The Venn diagram illustrated the predicted target genes from miRDB, TargetScan, and miRTarBase. The overlaps represented the numbers of genes predicted by more than one database (**a** miR-34a-5p; **b** miR-410-3p; **c** miR-6720-3p)

**Fig. 9:**
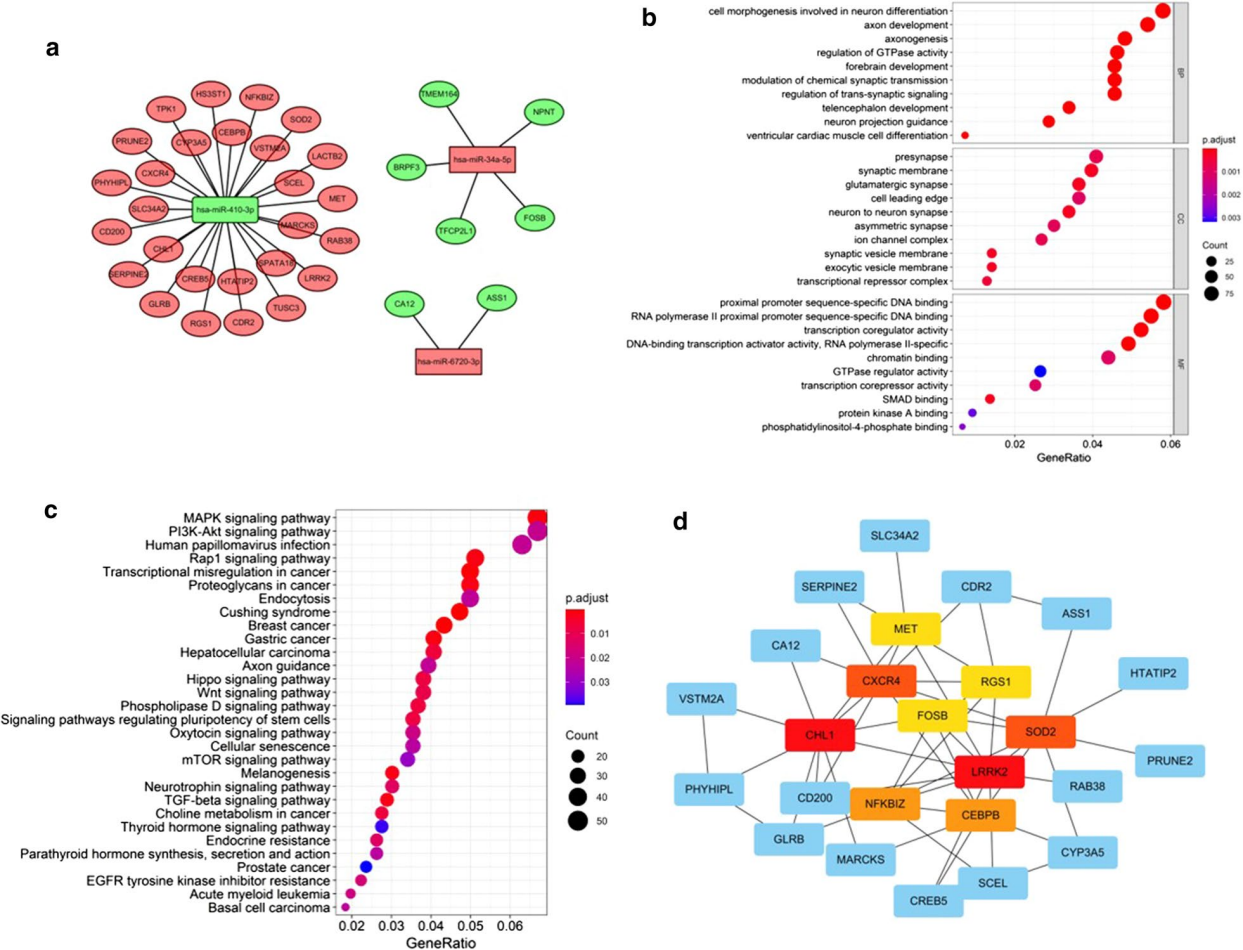
The regulatory and interaction networks between sDEmiRs and target genes, and functional enrichment analysis. The regulatory network of sDEmiRs and target genes (**a**); red parts represent up-regulation and green parts represent down-regulation. The top pathways of target genes are illustrated in biological process, cellular component, molecular function (**b**), and KEGG pathway (**c**). Protein–protein interaction network of target genes revealed their potential relationships (**d**)

In order to further explore the underlying regulatory relationships between sDEmiRs and their target genes, we first predicted the target genes by the databases of TargetScan, miRTarBase and miRDB, and the predicting results were illustrated in the Venn diagrams (Fig. 8a–c). Besides, the regulatory networks among the three sDEmiRs and their target genes were displayed in Fig. 9a. Because of these target genes also had the significant correlation with OS, we further detected the survival curve of these target genes. We found that the higher expression of SLC34A2, SPATA18, TPK1, CHL1, LRRK2, PHIHIPL and SCEL were related with the poor prognosis, while the higher expression of TUSC3, TMEM164 and CEBPB were correlated with the longer OS (Additional file 3: Figure S2). Functional enrichment analysis was performed through the Gene Ontology (GO) and Kyoto Encyclopedia of Genes and Genomes (KEGG) pathways to explore the potential molecular mechanisms of target genes. The functional enrichment analysis results of the target genes illustrated that “cell morphogenesis involved in neuron differentiation”, “presynapse” and “proximal promoter sequence-specific DNA binding” were the most enriched terms in biological processes (BP), cellular components (CC) and molecular functions (MF), respectively (Fig. 9b). “MAPK signaling pathway” was confirmed to be the most enriched among the KEGG pathway of target genes (Fig. 9c). To further explore the interactions of these target genes, we applied protein–protein interaction (PPI) network analysis, and the results showed that CHL1, LRRK2, MET, SOD2, CXCR4, CEBPB, NFKBIZ, FOSB and RGS1were the core genes among the target genes (Fig. 9d).

### MiR-410-3p and miR-6720-3p were overexpressed and miR-34a-5p was down-regulated in pRCC patients especially with advanced T-stages

**Fig. 10:**
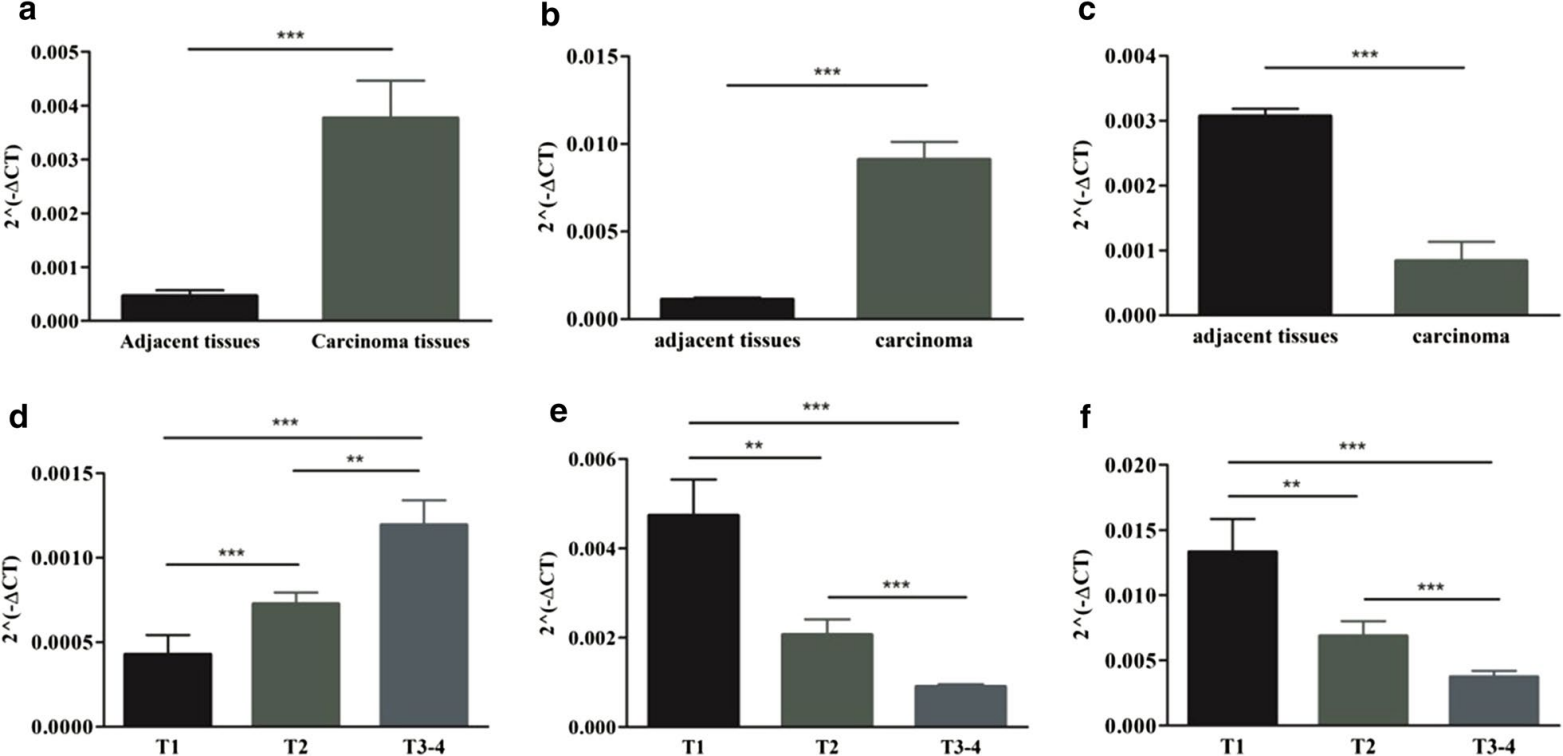
The expression levels of sDEmiRs and their correlations with T-stages. The expression levels of miR-410-3p (**a**), miR-6720-3p (**b**) and miR-34a-5p (**c**) in pRCC tumor and adjacent tissues based on the pRCC samples of The Affiliated Hospital of Southwest Medical University. The correlations of miR-410-3p (**d**), miR-6720-3p (**e**) and miR-34a-5p (**f**) with various T-stages based on the pRCC samples of The Affiliated Hospital of Southwest Medical University. The data are expressed as the mean ± SD. ***P < 0.001, **P < 0.01, *P < 0.05

To further validate the predicting effects of sDEmiRs on clinical prognosis of pRCC and the correlations between sDEmiRs and the clinicopathological parameters, we examined the expression levels of miR-410-3p, miR-6720-3p and miR-34a-5p in tumor and adjacent tissues of pRCC patients with different T-stages. MiR-410-3p (Fig. 10a) and miR-6720-3p (Fig. 10b) showed significantly higher expression levels in tumor tissues than those in adjacent tissues, however miR-34a-5p (Fig. 10c) illustrated the inversed change. Besides, compared with pRCC patients of T1 and T2 stages, the obviously less miR-34a-5p (Fig. 10d) and the remarkably higher expression levels of miR-410-3p (Fig. 10e) and miR-6720-3p (Fig. 10f) were detected in carcinoma tissues of T3-4 stages.

## Discussion

Although surgery and some replacement therapies including immunotherapy have been widely applied for pRCC patients, the limited response rate and unsatisfied outcomes motivated us to further explore the more appropriate methods to improve the therapeutic efficiency and achieve more personalized treatment from the perspective of predicting prognosis [21–23]. Because of the significantly individual variation, a class of genetic markers including coding and non-coding genes attracted increasing attentions in the past few years [24–26]. Liu et al. indicated lncRNA KTN1-AS1 played the remarkable roles on predicting the poor prognosis of non-small cell lung cancer and facilitate tumor progression through regulating miR-23b/DEPDC1 axis [27]. Zhou et al. developed a profile of TILBlncSig consisting of eight long non-coding RNAs (lncRNAs) identified from 141 B cell specific lncRNAs and demonstrated the potential implications in prognosis of bladder cancer [28].

With the more distinct advantages of the relatively stable state in tissues and the prominent testability, miRNAs become increasingly promising to be indicators to forecast prognosis [29, 30]. Gluud et al. demonstrated the indicating roles of miR-223, miR-191 and miR-342 on therapeutic outcomes of cutaneous T cell lymphomas [31]. Roth et al. found the miR-10b, miR-34a and miR-155 in serum of patients with breast cancer could be biomarkers for metastasis [32]. Besides, miRNAs in exosomes have also been identified to participate in the progression and prognosis of various tumors. Zhang et al. ascertained the miR-200c and miR14 in exosomes were the early biomarkers for metastasis of breast cancer [33]. Accumulating evidence highlighted numerous miRNAs were involved in the oncogenic survival pathways and closely associated with the progression and prognosis of tumors [34]. Efforts to further develop predictive miRNA profiles, and elucidate and corroborate more miRNAs that are potential for predicting prognosis will pave the way for the improvement of treatment efficacy and the implement of individualized medicine.

In the present study, we analyzed mRNAs and miRNAs data of 321 patients with pRCC in TCGA, of which 782 mRNAs and 164 miRNAs were up-regulated, and 462 mRNAs and 142 miRNAs were down-regulated. Subsequently, we identified 18 DEmiRs that were significantly correlated with OS (sDEmiRs), of which three sDEmiRs were further selected to establish the RSM which could be served as an independent prognostic factor to evaluate the prognosis of patients with pRCC. In the RSM, the younger patients, and patients with advanced stage, advanced T-stage, advanced N-stage and advanced M-stage may get the higher risk scores. In the analysis of the regulatory networks of target genes of the three sDEmiRs, we found the lower expression levels of SLC34A2, SPATA18, TPK1, CHL1, LRRK2, PHIHIPL and SCEL were related with the longer OS, while the lower expression of TUSC3, TMEM164 and CEBPB were correlated with the poor prognosis. Besides, during the PPI analysis, nine target genes were regarded as the core genes.

To further improve the reliability and persuasion for providing clinical decisions, we recruited a considerable number of patients with pRCC. Furthermore, certain specific sDEmiRs (miR-410-3p, miR-6720-3p and miR-34a-5p) with obvious clinical significances have also been validated to be implicated in the prognosis of pRCC and potentially serving as the molecular bioindicators for forecast and assessment of OS. The further exploration of target genes of these sDEmiRs provided a novel insight for future researches.

Although the potency of sDEmiRs for predicting prognosis has been ascertained, and we also validated the expression levels of miR-410-3p, miR-6720-3p and miR-34a-5p in tumor tissues with different T-stages, some defects are still needed to be pointed out and further discussed. Firstly, the absence of the combination with the proteomics and metabolomics decreased the integrity. Additionally, the practical applying values of the RSM based on the three sDEmiRs have yet be adequately elucidated and on desperate need of wide corroboration. Thirdly, except for miR-410-3p, miR-6720-3p and miR-34a-5p, other potential sDEmiRs and their target genes as well as the correlated underlying mechanisms remain to be further explored. Fourthly, we didn’t distinguish the type 1 or 2 papillary RCC. Fifthly, we didn’t split the data into training and testing set because of limited sample size.

## Conclusion

In the present study, we comprehensively analyzed and validated the effects of sDEmiRs on predicting prognosis of pRCC. The results pave the avenue for establishing and optimizing a reliable and referable risk assessing model and provide novel insight into the researches of biomarkers and clinical treatment strategies.

## Supplementary information

**Additional file 1: Table S1.** The results of multivariate Cox regression coefficients.

**Additional file 2: Figure S1.** The relationship between the hsa-miR-34a-5p and age. The younger patients were correlated with the lower expression levels of miR-34a-5p.

**Additional file 3: Figure S2.** Survival curve of target genes. Kaplan‐Meier survival curve of target genes. The higher expression of CEBPB (A), TMEM164 (B) and TUSC3 (C) were correlated with the poor prognosis. The higher expression of CHL1 (D), LRRK2 (E), PHYHIPL (F), SCEL (G), SLC34A2 (H), SPATA18 (I) and TPK1 (J) were associated with the longer OS.

## Data Availability

Authors can provide all of datasets analyzed during the study on reasonable reques**t.**

## Abbreviations

DEmiRs: Differentially expressed miRNAs
FDA: Food and Drug Administration
GO: Gene Ontology
GSEA: Gene set enrichment analysis
HR: Hazard ratio
KEGG: Kyoto Encyclopedia of Genes and Genomes
lncRNAs: Long non-coding RNA
mRNAs: Messenger RNAs
miRNAs: MicroRNAs
OS: Overall survival
pRCC: Papillary renal cell carcinoma
PCA: Principal component analysis
PPI: Protein protein interaction
qPCR: Quantitative polymerase chain reaction
RCC: Renal cell carcinoma
RSM: Risk score model
sDEmiRs: Survival related DEmiRs
TCGA: The Cancer Genome Atlas
TME: Tumor microenvironment
