# Supplementary material for: Identification of a three-miRNA signature as a novel prognostic model for papillary renal cell carcinoma
Source: Cancer Cell Int. 2020 Jul 16;20:317. doi: 10.1186/s12935-020-01398-2 (PMC7367267; doi:10.1186/s12935-020-01398-2)
Supplement: Supplementary file 1 — Additional file 1: Table S1. The results of multivariate Cox regression coefficients. [file 12935_2020_1398_MOESM1_ESM.docx]

**Additional file 1: TableS 1: The results of multivariate Cox regression coefficients**

| Gene | Coefficients | HR | HR.95% low | HR.95% high | *P* value |
| --- | --- | --- | --- | --- | --- |
| hsa-miR-34a-5p | -0.91267 | 0.40145 | 0.301975 | 0.533693 | 3.34E-10 |
| hsa-miR-409-5p | 0.034603 | 1.000035 | 1.000015 | 1.000054 | 0.059126 |
| hsa-miR-495-3p | 0.060359 | 1.062218 | 0.989387 | 1.14041 | 0.095803 |
| hsa-miR-379-5p | 0.082181 | 1.085652 | 0.986722 | 1.194501 | 0.091842 |
| hsa-miR-381-3p | 0.131079 | 2.254905 | 1.071294 | 4.746221 | 0.132248 |
| hsa-miR-105-5p | 0.185417 | 1.20372 | 1.059373 | 1.367735 | 0.244199 |
| hsa-miR-323a-3p | 0.196573 | 1.217224 | 0.949729 | 1.560059 | 0.120523 |
| hsa-miR-519a-5p | 0.200574 | 1.222104 | 1.039827 | 1.436333 | 0.149382 |
| hsa-miR-216a-5p | 0.203263 | 1.225395 | 0.997102 | 1.505957 | 0.053313 |
| hsa-miR-376c-3p | 0.261415 | 0.769962 | 0.545561 | 1.086662 | 0.136974 |
| hsa-miR-6720-3p | 0.271949 | 1.312521 | 1.016667 | 1.694469 | 0.036906 |
| hsa-miR-411-3p | 0.349336 | 2.547044 | 1.38213 | 4.693797 | 0.272145 |
| hsa-miR-539-5p | 0.422589 | 1.525907 | 1.070067 | 2.175931 | 0.19596 |
| hsa-miR-410-3p | 0.456773 | 1.57897 | 1.210824 | 2.05905 | 0.000745 |
| hsa-miR-337-3p | 0.604245 | 1.82987 | 1.333535 | 2.510938 | 0.181906 |
| hsa-miR-382-5p | 0.63326 | 0.530859 | 0.317803 | 0.886747 | 0.155579 |
| hsa-miR-224-5p | 0.648073 | 0.523053 | 0.29158 | 0.938281 | 0.297326 |
| hsa-miR-493-3p | 0.694574 | 2.002855 | 1.292645 | 3.103271 | 0.187789 |

Note: HR: Hazard Ratio.
