# Supplementary figures and images for: Identification of a three-miRNA signature as a novel prognostic model for papillary renal cell carcinoma
Source: Cancer Cell Int. 2020 Jul 16;20:317. doi: 10.1186/s12935-020-01398-2 (PMC7367267; doi:10.1186/s12935-020-01398-2)

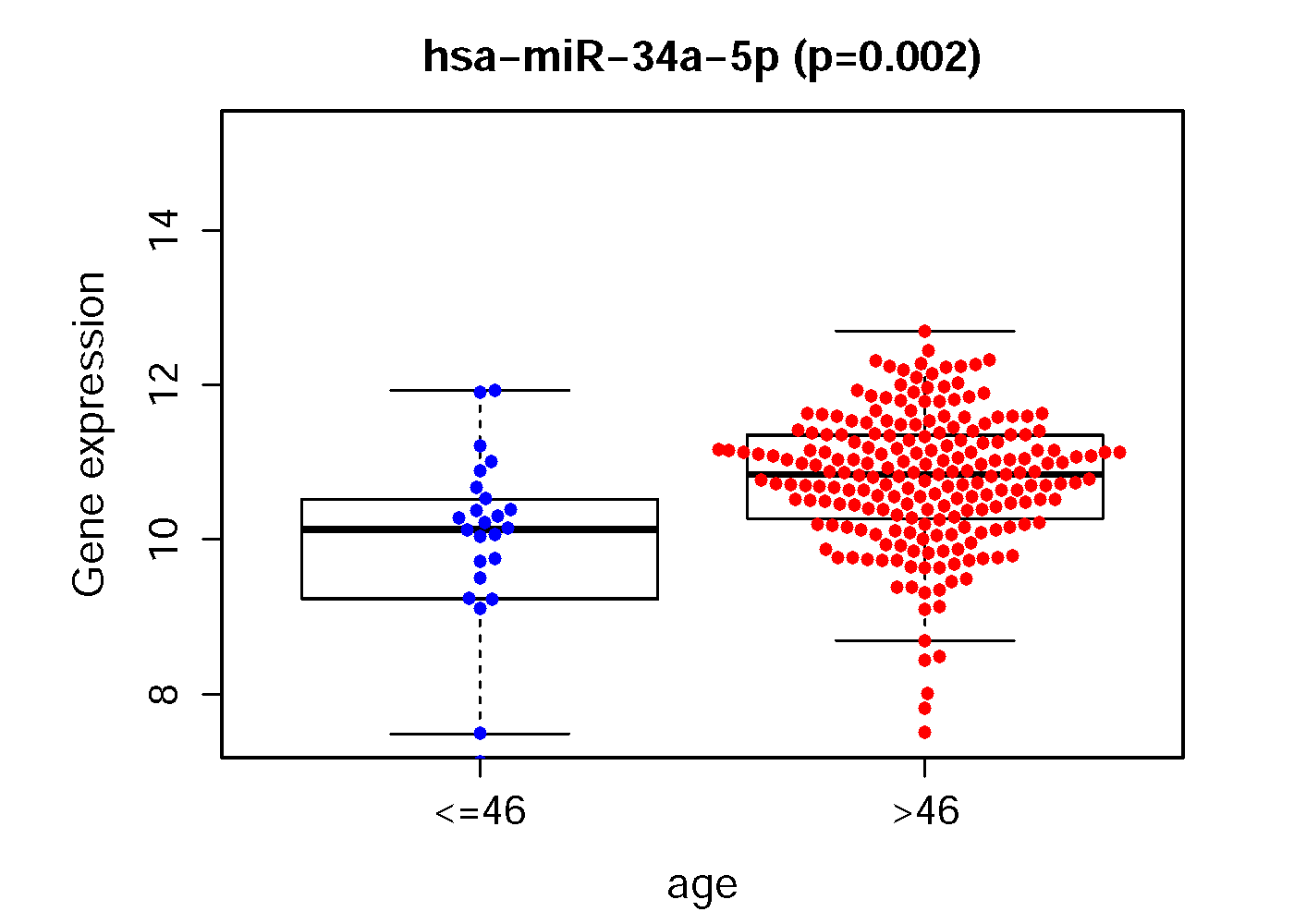

Supplement: Supplementary file 2 — Additional file 2: Figure S1. The relationship between the hsa-miR-34a-5p and age. The younger patients were correlated with the lower expression levels of miR-34a-5p. [file 12935_2020_1398_MOESM2_ESM.tiff]

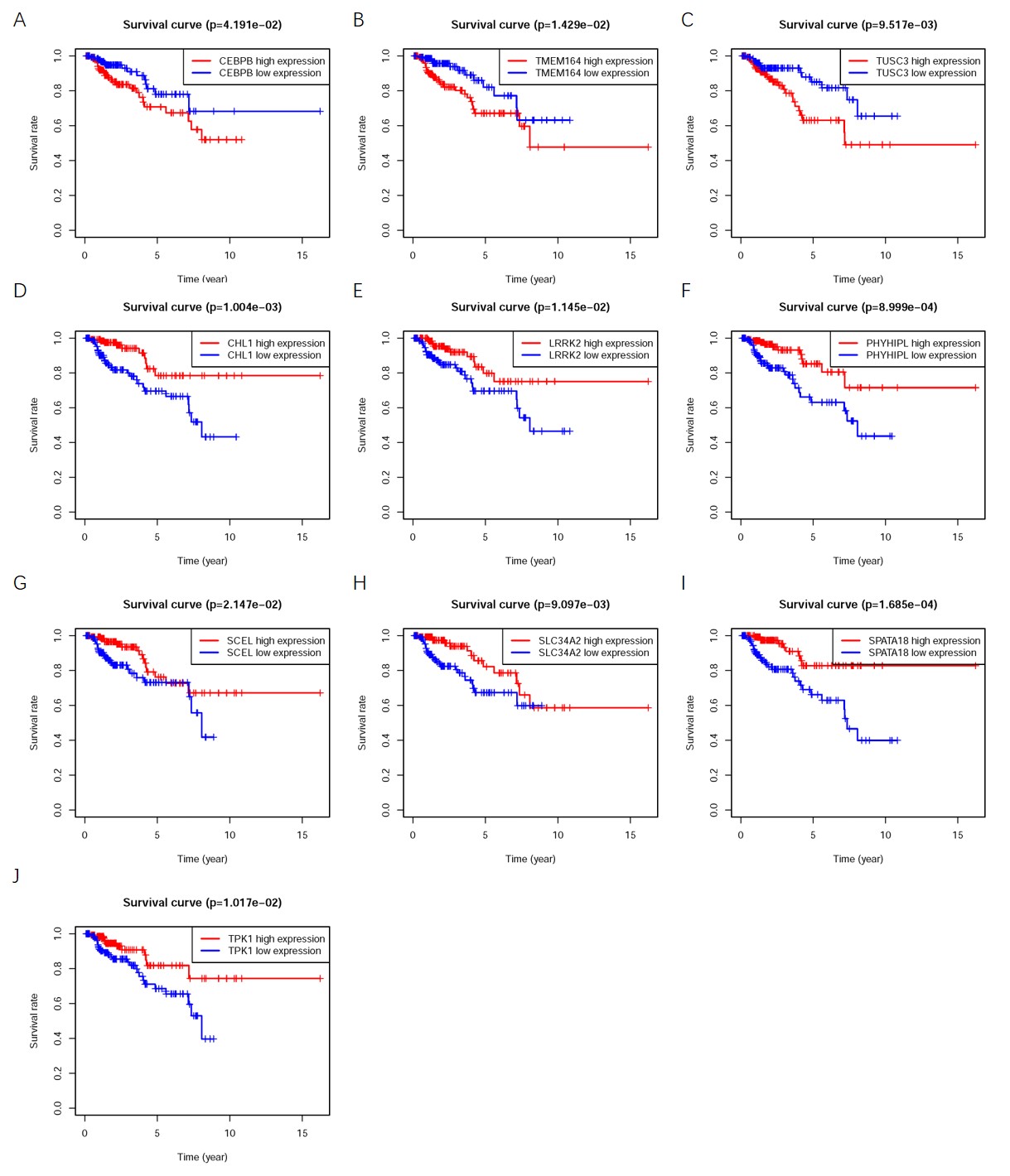

Supplement: Supplementary file 3 — Additional file 3: Figure S2. Survival curve of target genes. Kaplan‐Meier survival curve of target genes. The higher expression of CEBPB (A), TMEM164 (B) and TUSC3 (C) were correlated with the poor prognosis. The higher expression of CHL1 (D), LRRK2 (E), PHYHIPL (F), SCEL (G), SLC34A2 (H), SPATA18 (I) and TPK1 (J) were associated with the longer OS. [file 12935_2020_1398_MOESM3_ESM.jpg]
